# The *Virtual-Spine* Platform—Acquiring, visualizing, and analyzing individual sitting behavior

**DOI:** 10.1371/journal.pone.0195670

**Published:** 2018-06-13

**Authors:** Stephen Jia Wang, Björn Sommer, Wenlong Cheng, Falk Schreiber

**Affiliations:** 1 Department of Innovation Design Engineering, School of Design, Royal College of Art, London, United Kingdom; 2 International Tangible Interaction Design Lab, Monash University, Melbourne, Victoria, Australia; 3 Department of Computer and Information Science, University of Konstanz, Konstanz, Baden-Württemberg, Germany; 4 Faculty of Information Technology, Monash University, Melbourne, Victoria, Australia; 5 Faculty of Engineering, Monash University, Melbourne, Victoria, Australia; University of Illinois at Urbana-Champaign, UNITED STATES

## Abstract

Back pain is a serious medical problem especially for those people sitting over long periods during their daily work. Here we present a system to help users monitoring and examining their sitting behavior. The Virtual-Spine Platform (VSP) is an integrated system consisting of a real-time body position monitoring module and a data visualization module to provide individualized, immediate, and accurate sitting behavior support. It provides a comprehensive spine movement analysis as well as accumulated data visualization to demonstrate behavior patterns within a certain period. The two modules are discussed in detail focusing on the design of the VSP system with adequate capacity for continuous monitoring and a web-based interactive data analysis method to visualize and compare the sitting behavior of different persons. The data was collected in an experiment with a small group of subjects. Using this method, the behavior of five subjects was evaluated over a working day, enabling inferences and suggestions for sitting improvements. The results from the accumulated data module were used to elucidate the basic function of body position recognition of the VSP. Finally, an expert user study was conducted to evaluate VSP and support future developments.

## 1. Introduction

Musculoskeletal back pain is a known consequence of incorrect posture and prolonged muscular inactivity (i. e., being stationary at one point for a lengthy period). Back pain is the number one cause of global disease burden worldwide [1], and musculoskeletal disorders such as lower back pain and osteoarthritis are now second only to cancer as the leading cause of disease burden in Australia. The time spent sitting is associated with premature mortality, diabetes, and risks of cardiovascular disease [1, 2], irrespective of time spent exercising [3]. Given that approx. 80 per cent of Australians suffer chronic back pain at some stage in their lives with an economic cost of USD 9.17 billion per year, the impact and commercial potential to improve sitting behaviors in people’s daily life are enormous. Sitting-related back pain especially affects those who sit at work. An international epidemiological study on about 50,000 adults reported sitting time was 300 minutes/day on average [2].

A number of studies have convincingly reported the association between different levels of exposure to occupational sitting and the presence or severity of low back pain [3]. There is also unequivocal evidence that sitting and upper quadrant musculoskeletal pain are related [4]. It is a challenge to maintain appropriate sitting positions in daily life to avoid seating-related health issues. In the literature discomfort and pressure sores have received particular attention in the military [5], workplace [6], assisted living [7, 8] and mobility [9–11] contexts. For instance, the findings of Burnett et al. articulate the challenge to maintain appropriate sedentary behavior [12]. Despite the controversy around what constitutes an ideal sitting posture, it is clear that sitting in fixed positions, particularly for prolonged periods of time, significantly increases one’s risk of developing lower back pain due to the static loading of soft tissues and discomfort [13]. Since prevention is better than cure, precautions are required to circumvent posture-related back pain.

The chronological sequence of individual sitting postures during the day is as unique as a fingerprint. While a common ergonomic chair might encourage standardized postures [14–16], it does not consider user’s individual body needs and unique situations; and it also cannot provide feedback to the user to adjust the body position. Spinal radiography provides some measurable parameters of spinal curvature in the clinic environment [6, 7]. However, the exposure to constant radiation is not a real-life solution for an average office worker. Following the development of embedded sensing technologies, a trend is emerging in the smart furniture industry to utilize this technology for user-centered health applications. For example, large and flexible tactile sensors allow examination of pressure distributions and enable quantitative evaluation of the pressure-affected comfort of a seat cushion [17, 18]. Real time body position recognition can now be realized [19] and sensors can sense the weight of an occupying item on a seat [20]. Real-time sensing data from smart furniture can now also be wirelessly monitored with smart phones [21]. However, merely sensing pressure distribution and body movement cannot provide adequate spine health-related behavioral support. This data must be combined with accurate spine movement patterns and personalized user information to enable the success of a sophisticated health support system.

The technologies currently available for spine position recognition in the laboratory environment, operative environment, and daily living environment are outlined below:

1. In the laboratory: in ergonomics research (especially occupational research settings) popular non-invasive and comparatively economical methods utilize surface markers [22], digital photography, or video analysis [23, 24] to analyze spinal postures. CODA™ is an example of a surface marker-based motion analysis system to detect lumbar spine sagittal plane range of movement and posture [25]. However, without direct skin contact, the sensitivity of such methods cannot provide adequate support to identify subtle spinal movements [26].
2. In the clinic: current methods to accurately monitor the in vivo spine situation involve radiography, electromyography and surface markers applied in clinical situations. Spinal cord monitoring [27–30] and intraoperative monitoring [31–33] are diagnostic procedures in which electrodes are applied to the skin and feedback is obtained assessing the health of muscles and the motor neurons controlling them. Unfortunately, these systems cannot provide posture and curvature information, are costly, complex, time-consuming and cannot be easily used in “real-life” settings and are limited by their inability to provide instantaneous postural feedback during daily tasks. This function is crucial, considering the harmful impact of reduced postural awareness may play in back pain [34]. Despite their potential, many of these devices are large and cannot be concealed easily [35], can only be used under supervision [36] or only provide a snapshot of static spinal posture without also analyzing dynamic posture or providing postural feedback [37, 38].
3. In real-life situations: there are only a few products in the current market able to provide upper body posture monitoring, such as Lumoback^®^ (Registered to zero2one, Palo Alto, California) and Darma^®^ (Registered to Kickstarter Inc., New York) for spine curvature monitoring or monitoring spinal motion [39] and BodyGuard™ which identifies lumbo-pelvic posture and movement [40] via detecting spinal sagittal plane posture [26]. Spineangel^®^ [28] provides trunk flexion such as hip, lumbar and total sagittal rotation and pelvic tilt in real-time via 3D motion analysis. Although these methods provide monitoring of spine movement, they all require certain body-attachment, which is impractical and sometimes very time consuming. Also, most of these devices cannot provide personalized support.

Therefore, there is an urgent need to develop a method which can provide accurate, personalized sitting behavior support without annoying the user, and encompass a pervasive computing approach. In this work, we outline how the aforementioned problems can be overcome through noninvasive monitoring of spinal movements whilst sitting and how it can be immediately fed back to the user.

## 2. Materials and methods

**Fig 1:**
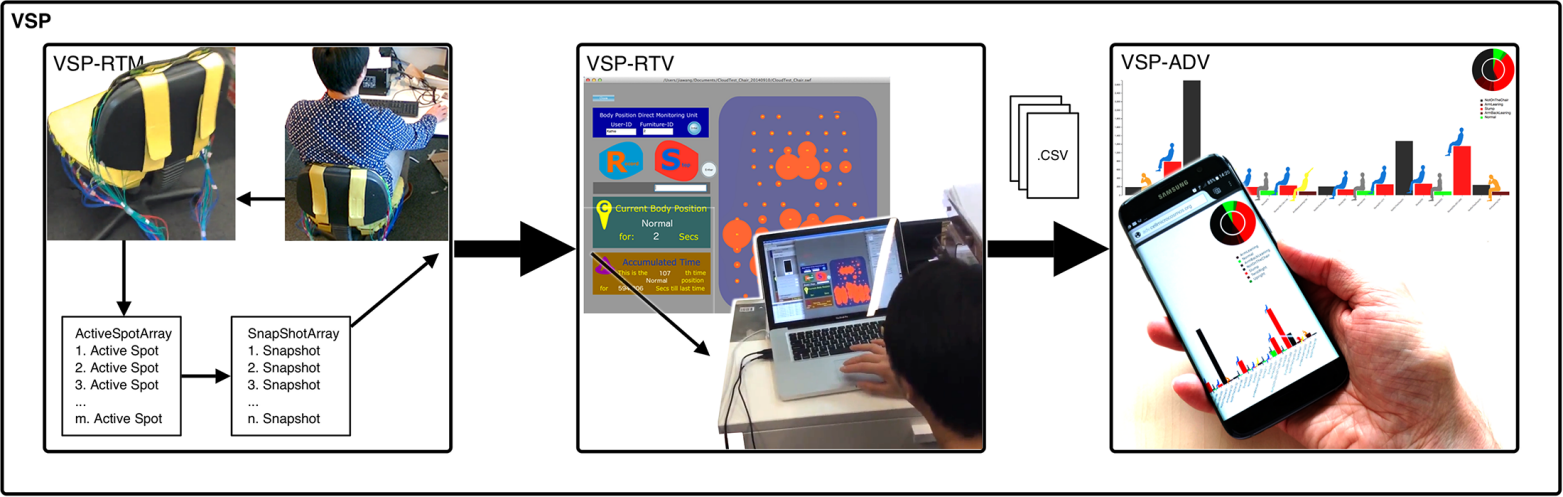
Virtual-Spine system overview. Left: VSP-RTM: Real-Time Monitoring system; Center: VSP-RTV: Real-Time Visualization system; Right: VSP-ADV: Accumulated Data Visualization system.

Here we present a prototype which can be used for non-invasive identification and analysis of spinal movements, enabling medical experts as well as patients to interpret the data. The *Virtual SPine system (VSP)* consists of the following three major components (see Fig 1):

1. *VSP-RTM—Real-Time Monitoring system* (2.3 Recognition of Sitting Postures)
2. *VSP-RTV—Real-Time Visualization system* (2.4 Real-time Data Visualization)
3. *VSP-ADV—Accumulated Data Vis. system* (2.5 Accumulated Data Visualization)

### 2.1 Design of the Virtual-Spine Platform (VSP)

**Fig 2:**
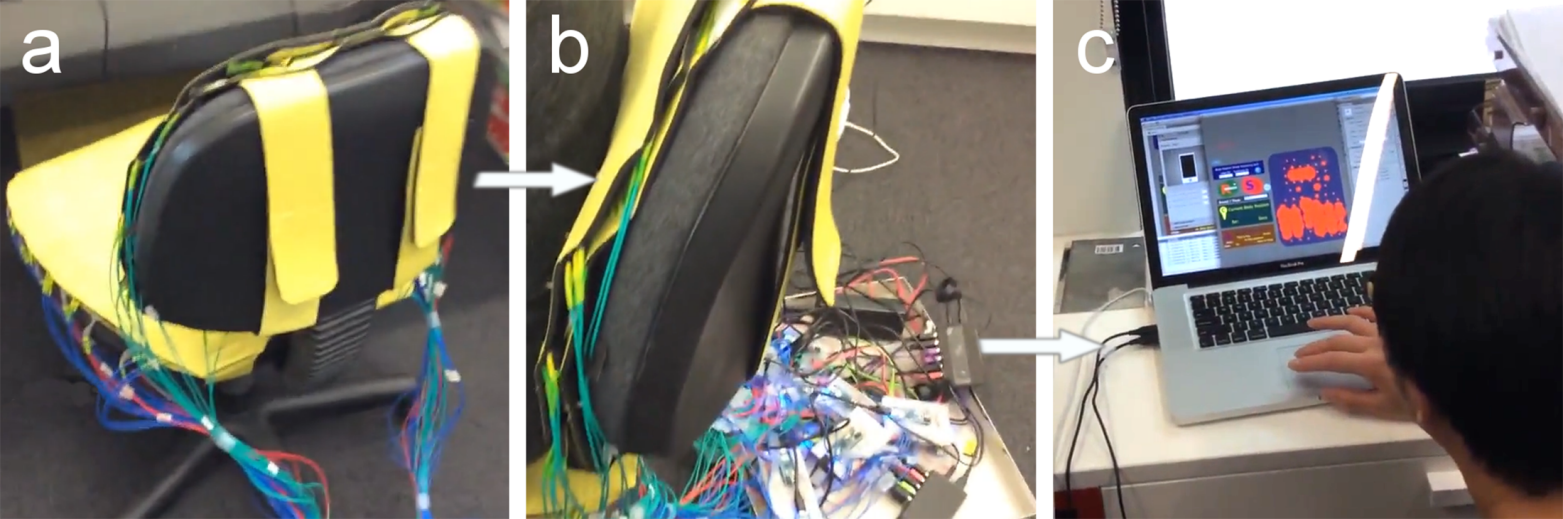
Virtual-Spine: Real-time monitoring and visualization system. The Real-time Monitoring System: a) in combination with a conventional office chair showing the VSP back and seat mats attached with sensors; b) the connecting hardware of the VSP mat to the computer system; The Real-time Visualization System: c) a laptop running the posture recognition system (see also Fig 4).

This work applies a novel pervasive monitoring system, the *Virtual-Spine Platform (VSP)* [41] to prevent fixed and harmful postures in prolonged sitting without attaching any additional equipment to the user’s body. The aim of “Virtual-Spine” is to profile users’ spinal movements in the daily life environment. For this purpose, a comprehensive agent model was developed, intends to gather information from various types of chairs in multiple locations (e. g. office chairs, sofas, dinning chairs, etc.) which are equipped with a VSP smart-mat (see Fig 2b). Unlike portable monitoring devices which either monitor body posture or provide postural feedback, VSP is able to support future smart chair devices to monitor spinal alignment (instead of body postures) and provide immediate postural feedback to the user without the need for monitoring equipment attached to the body.

VSP is designed to constantly monitor everyday activities of users by using the *Real-Time Monitoring module (VSP-RTM)* [41, 42] (see also Fig 2). During the experiment, data providing an overview of a user’s sitting behavior in a certain period is accumulated and stored for analysis. The applied visualization methods enable multiple users to simultaneously view, discuss, and interact with the spine profile, supporting remote and co-located activities [43]. Although the experiment discussed in this paper is targeting regular users working in an office-like environment, VSP is also a promising approach for doctors, health advisors and personal trainers to support their health practices with both real-time and accumulated spinal alignment (potentially pressure load) information. In this way, the experts could use this information to provide patients with suggestions for changing or optimizing their sitting behavior. The methods could also be used to compare sitting behavior before and after the treatment. In the context of this work, the term *Smart Chair* describes a regular office seat combined with the matte connected to the VSP-RTM system, as seen in Fig 2.

Fig 2a shows the VSP prototype in action. The system is featured with a *sensory unit* (Fig 2a/2b) which is designed to monitor the user’s body positions using a matrix of pressure sensors that fit on the furniture occupied by the individual. It can be designed in suitable forms and set up in various places to gather sedentary position information in different contexts [44], such as the office, at home, or during driving. In this work, the sensory unit consists of 68 force-sensitive resistors, 26 at the back, and 42 at the seat, as seen in Fig 2a/2b. For the back mat, the distance between sensors is 70mm (Height) and 60mm (Width), whereas the top and bottom lines are 85mm. For the seat mat, the distance of each sensor was 50mm (H) and 70mm (W), with a slightly wider distance of 115mm in the middle of the mat, which divided the mat sensing focus into both two sides of human bottom and thighs. The sensing matrix components in both mats were hand-made in the International Tangible Interaction Design Lab by using the Velostat/Linqstat Pressure-Sensitive Conductive Sheet from 3M.com^™^ with volume resistivity < 500 ohm-cm and surface resistivity: < 31,000 ohms/sq cm to achieve the resistance changes according to the pressures applied on each sensor.

To provide personalized information, the recorded data is a combination of:

1. Sensor data: recorded by the sensory unit, is required to compute the postures.
2. Chair-id: each Virtual-Spine-enabled “smart furniture” has a unique id, used to recognize which chair the user is sitting on, enabling contextual hypotheses. However, in this work, only a single chair is used.
3. Personal information:
  - user’s total sitting time over a certain period,
  - prolonged sitting time (over the limited time for particular sitting positions),
  - characteristics in user’s profile, e. g. age, BMI, medical history, and
  - rates of time sitting in appropriate (healthy) postures.

This combined data is sent to the “Advisory Unit” which processes it to compute the cumulative spinal burden.

### 2.2 Variation of sitting postures

Given the different and significant impacts on the spine alignments from various common sitting positions, firstly the system need to distinguish various basic sitting postures. It would be ideal to support users with “healthy” postures recommendations, however, there is no common understanding of a “good” or “bad” postures, but little quantitative basis can be referenced to define these postures [45]. Below we discuss an alternative approach to visually encode and judge the health-promoting effect of certain sitting behavior.

**Fig 3:**
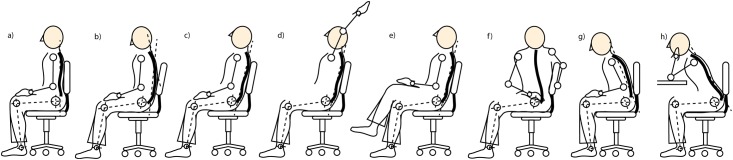
Sitting postures. a) *Upright*; b) *Slump*; c) *Normal*; d) *ArmBackLeaning*; e) *RightOverLeft* (or the opposite: *LeftOverRight*); f) *TwistLeft* (or the opposite: *TwistRight*); g) *PokingChin*; h) *ArmLeaning*.

It is also necessary to distinguish when a user is leaning towards different directions as well as the basic leg variations. Therefore, we considered eight main sitting positions during the development of VSP as shown in Fig 3a–3h:

- a) *Upright* (sitting straight up),
- b) *Slump* (slouching, leaning slightly forward),
- c) *Normal* (relaxed), and
- d) *ArmBackLeaning* (lean towards back).
- e) *RightOverLeft* (or the opposite: *LeftOverRight*),
- f) *TwistLeft* (or the opposite: *TwistRight*),
- g) *PokinChin* (lean towards front), and
- h) *ArmLeaning*.

### 2.3 Recognition of sitting postures

**Fig 4:**
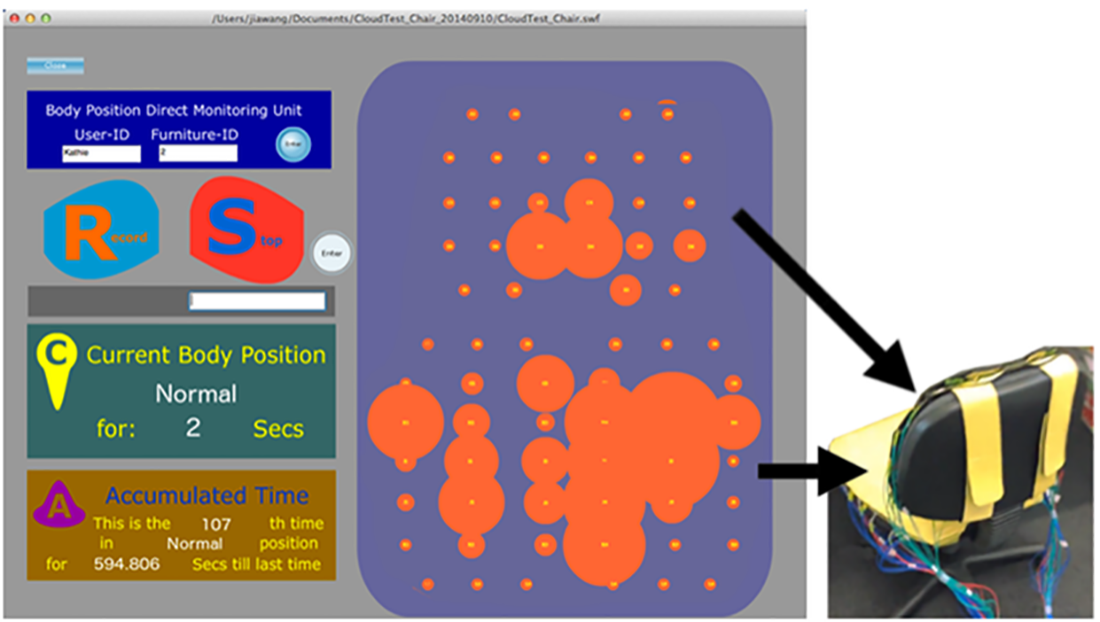
Real-time visualization module in VSP. a) Posture recording in an office environment, here, the posture *Normal* is shown (see also Fig 1 left and Fig 3c); b) Left: the posture-recording GUI and information of the current pose, Right: the pressure pattern of the *Normal* pose.

In the *Real-time visualization module* (VSP-RTM, see also Fig 1) the data of the sensory unit is computationally evaluated to interpret the corresponding actual user posture. The orange dots in the right blue area of Fig 4 represent the real-time readings of the 68 sensory units. The size of the dots shows the intensity of the pressure on the chair. The left side of Fig 4 shows the data which will be exported to be visualized in Section 2.5 Accumulated Data Visualization.

Prior to the monitoring process which can be used to analyze the sitting behavior of a user, the postures have to be defined and evaluated during a training phase. In order to detect and distinguish various sitting positions during the training phase, the following four major steps have been implemented to record the input training values for each posture (see also the GUI in Fig 4):

1. Creating Training Input: the software contains a “Snap Array” which is storing each “active” sensor input and which will be used as a blue print to be compared with real-time input training values in the later stage.
2. Taking Snap Shots: in calibration mode, a snap shot of each defined body position (e. g. Sitting *Straight Up*, *Slump*, *Normal*) is taken and the corresponding sensor value is stored in the Snap Array.
3. Repeating Snap Shot: to take into account variations of the same body position and verify the calibration, it is necessary to repeat snap shots for *n* times for a single posture, as people will sit in slightly different positions still representing the same posture. To achieve appropriate average values, the empirical value of *n = 6* was determined during our experiments.
4. Finalization or Next Posture: in the last step the user has to finalize the training process and go to the monitoring process, or to repeat the calibration step for another body position.

In order to efficiently detect and distinguish various position of the user’s body, it is necessary to extract the calibrated value of *Active Spots* each second—representing the state of the active sensors at the actual time point—from the sensory unit before comparing it to the Snap Shot patterns (see also Fig 1). Due to the constant change from non-relevant sensing values, the concept of Active Spots has been created to filter noisy sensor interferences to enhance the system’s stability and accuracy in pattern recognition. Only these Active Spots will be included in the following analysis. Therefore, in order to select these Active Spots from the sensory unit, the following process needs to be followed: First, an array is created to store the current input value of each sensor locally or online in a file. Second, the current input sensor value is compared to the current value record in the sensor as follows: for each sensor—where *Vep* is the value in the existing patterns and *ThreKP* is the threshold of each sensing points—if the following equation is true, then pick the sensor as the Active Spot:

Vep≤ThreKP

In the analysis stage, we have used a k-Nearest-Neighbour-based machine learning algorithm to effectively recognize the sitting positions in real-time. The major purpose of this algorithm is to compute the similarity between the new input matrix and the recorded patterns. In order to determine the nearest particular pattern *Sim*_*NPP*_ to the new input matrix, the Euclidean distance between each new vector *Iv* and the nearest pattern vector *Pv* has to be computed, wherein

$$Sim_{NPP}=\sqrt{\sum {\left(Iv-Pv\right)}^{2}}$$

The resulting value is normalized by the following equation:

$$Sim_{NPPN}=\sum {\left(\frac{Iv-Pv}{MaxVsen-MinVsen}\right)}^{2}$$

In order to analyse the value for each sensor, the following procedure has to be executed:

if (variance(Array Pattern Input) == Threshold Pattern) {

 Pattern Sensor Value = mean (Array Pattern Input);

} else {

 Pattern Sensor Value = DefaultValue;

}

The projection of array data between the “Training Input” and “Snap Shot” can be estimated by Active Spots Difference for different body parts measuring the differences for the same position-sensing data of the same body parts (i. e. shoulder, back, waist). Finally, with the following equation the sensors turn to Active Spots:

$$\frac{\sum {\left(Iv-Pv\right)}^{2}}{\sum {\left(MaxVsen-MinVsen\right)}^{2}}\leq ValT$$

Here *Iv* is the current input value of the sensor and *Pv* is the pattern value which is determined through the *n* times-repeated “Snap Shot” process. *MaxVsen* is the maximum sensor value and *MinVsen* is the minimum sensor value, that indicate the range of the *Iv* and *Pv* value, i. e., the difference is standardized. *ValT* is an empirical value obtained via experiments. In case the value obtained via the above equation is smaller or equal to *ValT*, the user’s sitting position is the corresponding pattern position. In the case that different postures are recognized by this procedure, the posture with the highest similarity is defined as the active posture.

During body position shifting, the posture similarity is dynamically calculated against existing patterns: if a user’s position is shifting from position A to B, the similarity to A will decrease while the similarity to B will increase. Once the similarity of B becomes larger than A, the actually recognized body position by VSP is B.

To summarize, as one of the main features of the VSP system design, the “Snap Shot” process provides the possibility to define the blue print of particular sitting positions. Therefore, a large number of postures could be defined during the “snap shot” process. The real-time reading data in the later monitoring process is compared to these blue prints in order to achieve posture recognitions.

To effectively improve the sitting behavior it is important to communicate the real-time monitoring result with the users in an efficient, intuitive, and user-friendly manner. However, in order to use the validation result of the given body position for comparison and reviewing in the later stage, it is also necessary to store the validated data independently. The array pattern is recorded for each sensor to a local file or uploaded into the cloud.

The data is then visualized with two different methods which will be discussed in the following:

1. Real-time Data Visualization
2. Accumulated Data Visualization

### 2.4 Real-time data visualization

The GUI for the *Real-Time data Visualization* (VSP-RTV, see also Fig 1) has been designed in a simple graphical style. In Fig 4, the enlarged size of the dots illustrates the amount of the pressure which has been applied on the chair. The left side of Fig 4 indicates the functions of (in top-down order):

1. Setting up user’s ID and chair ID (blue colored area),
2. Record Snap Shot (light-blue button) and stop Snap Shot process (red button),
3. Current body position and recorded time period (green area),
4. The accumulated time depicts the current position, the duration of the period the user has been sitting in this certain position, and the accumulated seconds the user was in this position during a single experiment period.

In this experiment, we have chosen the following eleven postures for the system to differentiate: *ArmLeaning*, *ArmBackLeaning*, *LeftOverRight*, *Normal* (Fig 1a), *NotOnTheChair*, *PokingChin*, *RightOverLeft*, *Slump*, *TwistLeft*, *TwistRight*, *Upright*. Each of these postures is defined by a specific pressure pattern measured by the sensors. Fig 4 presents the real-time visualization method used to observe the data recording process. The right blue area shows the 2D mapping of pressure measurements. The software connects this pattern to a specific pose as shown on the green area on the left side. It also shows the time the user remained in this pose and the number of times this pose was repeated.

The Supporting Material provides a video showing the pose recording process by using the VSP-RTV, see Supporting Material S1 Video.

### 2.5 Accumulated data visualization

While the previously discussed visualization method (VSP-RTV) was used to identify and present different poses during the experiment, the following approach—the *Accumulated Data Visualization* (VSP-ADV, see also Fig 1)—is used to analyze the experiment’s results and to provide feedback and recommendations for the user according their sitting behavior. Whereas VSP-RTV could only be run on a local computer, the following system is developed to share the sitting postures online, enabling the comparison to other sitting behaviors and the discussion with distant experts. For this purpose, the data which contains all postures and their duration is imported and sitting behavior patterns are created.

A problem was to present the posture charts in a way which a) visualizes the postures in an easily-decodable fashion and b) follows Shneiderman’s visualization mantra [46]. To explain the design of the visualization system we follow Keim’s taxonomy [47].

#### Classification of data types

The following data types have to be taken into account:

- posture type (see Fig 3)
- time (see Fig 4)
  - posture starting time
  - posture duration

#### Classification of visualization techniques

**Fig 5:**
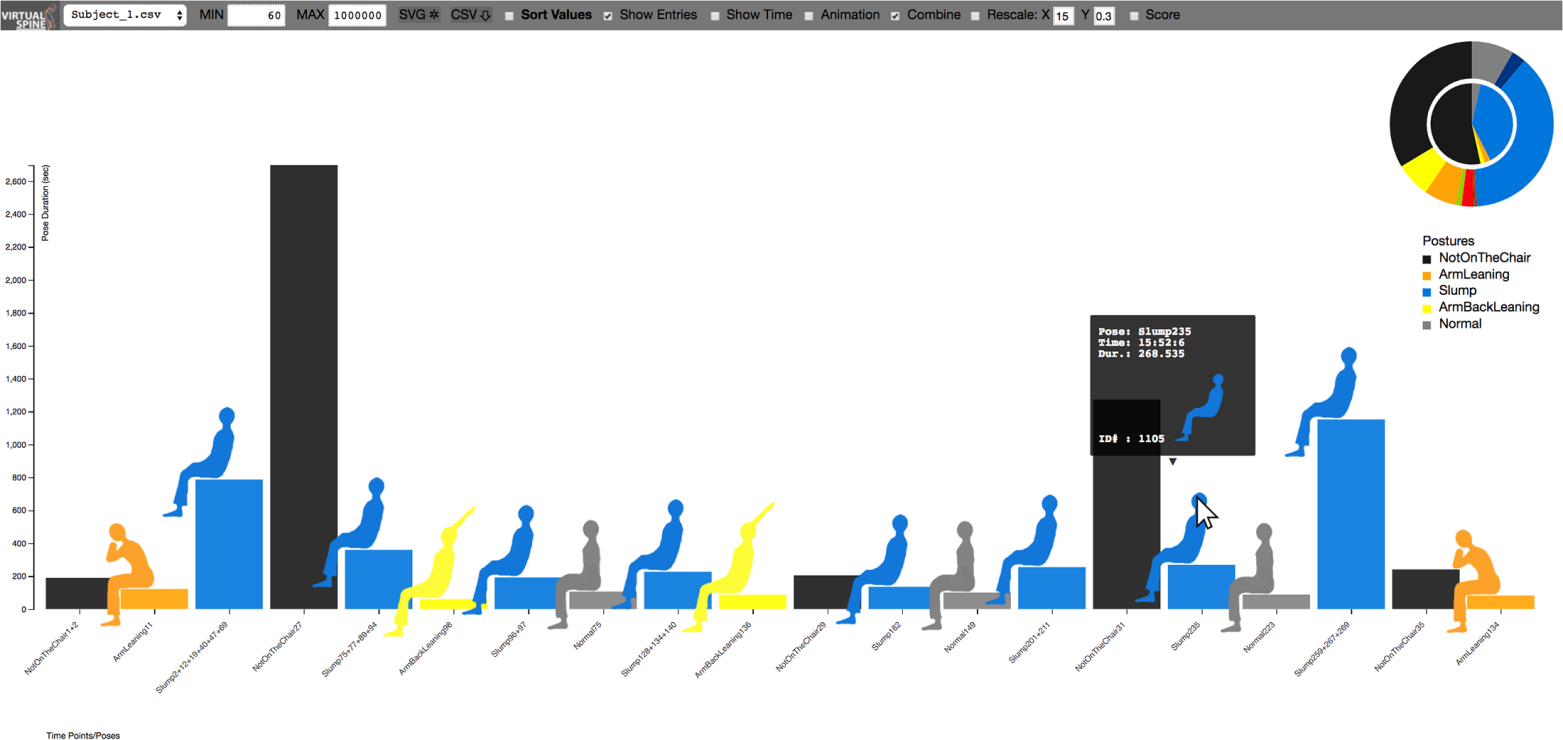
VSP-ADV with posture type-dependent coloring. The interactive pose bar charts are ordered based on their time sequence and only those poses are shown with a duration *d* of 60 *sec*. < *d* < 1,000,000 *sec*. The figure shows also the details on demand feature, providing information about the selected posture period. The X axis represents the posture time points following the filtering rule, whereas the Y axis represents the posture duration.

**Fig 6:**
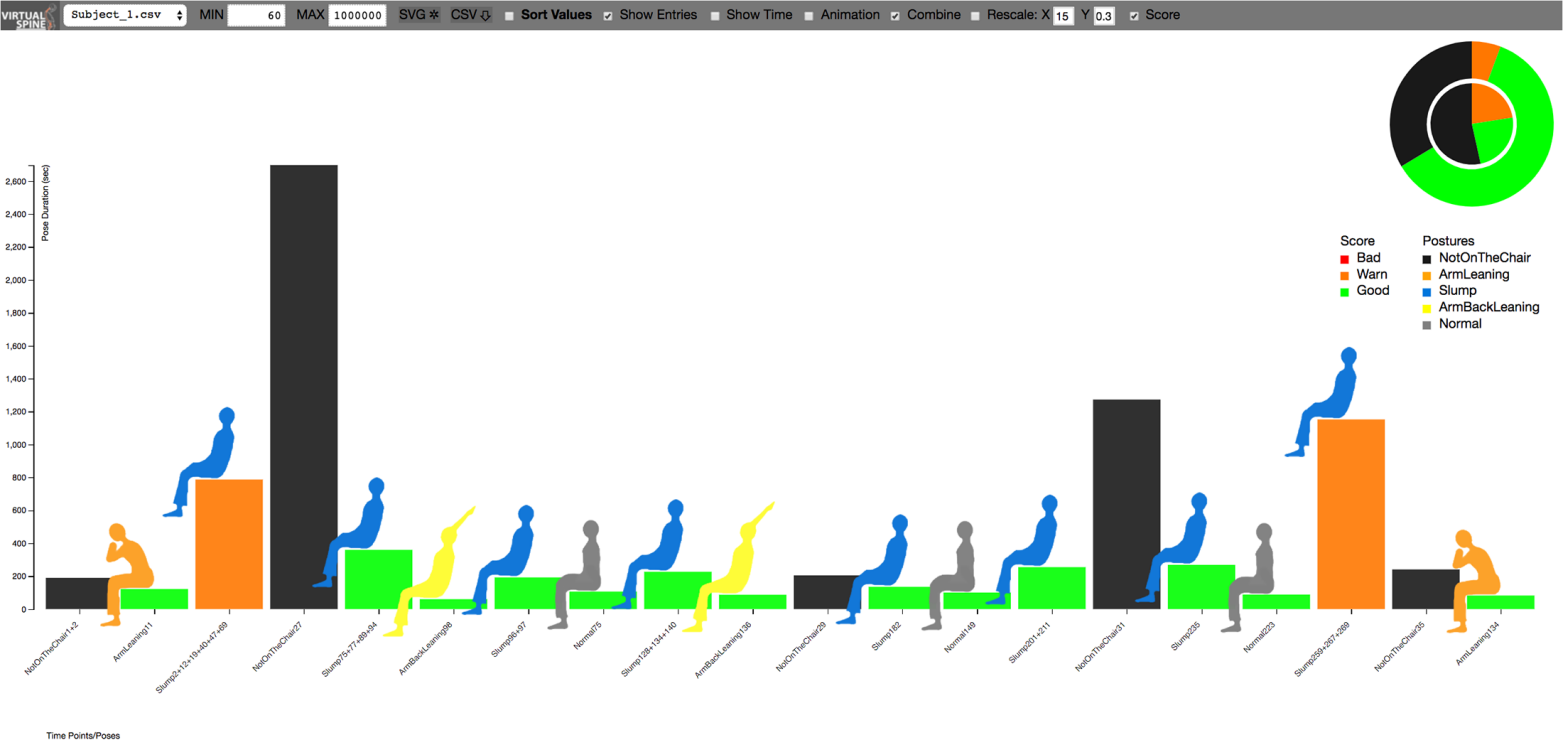
VSP-ADV with scoring posture-dependent coloring. Score colors, in this mode represented by the bars: Green: good with *d* ≤ 8 *min*., Orange: warn with 8 *min*. < *d* ≤ 20 *min*., Red: bad with 20 *min*. < *d*. Subject 1 showed a quite good sitting behavior, as no red warning is shown at all. Please see also the caption of Fig 5.

As the data type time contains two variables, namely the posture starting time and the corresponding duration, a standardized 2D display was used as the base for our visualization approach using bar charts and pie charts [48]. The bar chart represents the two dimensions of the time: the posture duration is presented along the Y axis, and the posture starting time along the X axis. The third dimension to be represented is the posture type. One approach to visualize the posture would be the use of a three-dimensional bar chart, using the Z axis for the posture type—each posture would be represented by another Z layer. To achieve a simple and focused presentation on a 2D display, we decided to color-code the different postures. To easily differentiate various postures, a color scheme based on the color alphabet was used [49–51]. This approach effectively supports the differentiation of the eleven variables discussed in the previous chapter. However, during first experiments, we experienced the difficulty to decode the posture types exclusively based on the color scheme. Although combined with a standard color scheme, it still demonstrated low-readability to normal users. Therefore, we decided to combine the simple 2D display/bar chart with an Iconic display [47]. Based on the posture types (Fig 3) we developed simplified icons/glyphs [48] of the different postures and combined them with the bar charts. In this way the readability of the bar chart was improved (see Figs 5 and 6).

The idea of combining charts and icons to visualize postures was used before, although—to our knowledge—never in a systematic and automatized way like in this approach. For instance, Nachemson used similar illustrations in studies on the pressure inside the low back intervertebral discs where he illustrated the difference in pressure distributions among different postures [52, 53]. Wilke et al. compared their results to the ones of Nachemson and used similar visualizations [54]. For commercial purposes similar images are used to explain the impact of back pain [55] or to discuss safe lifting techniques [56].

Although the combination of the two channels, color and glyph, improves the readability, the color channel can now also be used to encode alternative aspects. This approach enabled the visualization to address the previously mentioned idea to distinguish postures regarding to their health-promoting nature (Section 2.2 Variation of Sitting Postures).

For this purpose, we introduce a three-color-coded health rating scheme based on posture duration. Although there is no clear definition of “prolonged” sitting time, there is a small number of publications discussing the maximum time to remain in a single posture. In this study, we based our health rating on the following two research findings: first, Reenalda et al. concluded that the sitting posture should be changed at least every eight minutes [57]. Although the mentioned work had the main focus on wheelchair users evaluating the pressure distribution and oxygen tissue oxygenation during sitting, this recommendation will also hold for an optimal sitting behavior of non-disabled individuals. The second work taken into account is from Ryan et al. who examined the duration of sitting events [58]. 20 minutes of uninterrupted sitting time (i. e., without leaving the chair) where the lower bound used in that study, based on a recommendation for healthy subjects by the Chartered Society of Physiotherapy [59].

Based on these recommendations, the following health rating color-codes were introduced to VSP-ADV:

- good (green): *d* ≤ 8 *min*.
- warn (yellow): 8 *min*. < *d* ≤ 20 *min*. [57]
- bad (red): *d* > 20 *min*. [58, 59]

#### Classification of interaction and distortion techniques

The Accumulated Data Visualization module (VSP-ADV) is designed to visualize the accumulated readings over the complete time line. However, for the experiments discussed in the following sections, we were focusing on the analysis of postures with a minimum duration of 10 seconds; all lower durations are assumed as random movements on the chair. For this purpose, an interactive filtering technique was implemented [47] and we followed *Shneiderman’s visualization mantra* [46].

*1. Overview first:* first, the complete data set is shown. Whereas the standard setting of VSP-ADV uses already the 10 seconds filter for the bar chart, the global overview is provided by the pie chart: the outer pie chart is showing the summary of the complete data, whereas the inner pie chart is illustrating the filtered data with a minimum duration of 10 seconds.

**Fig 7:**
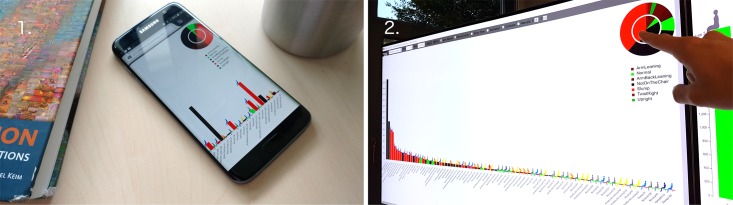
VSP-ADV on a smart phone and a high-resolution tiled display. During the development of VSP-ADV we were focusing on an application compatible with high-resolution displays (right) as well as smart phones (left).

*2. Zoom and filter:* the big advantage of a standard 2D display is the easy navigation by zooming and panning which can be used with standard monitors in combination with a computer mouse as well as touch display on mobile devices (Fig 7). On a high-resolution display panning will not be required to visualize the standard view, whereas on a mobile devices the option to zoom and pan is highly relevant. The interactive filter function is also provided. In this way it is possible to filter all postures by defining the minimum and maximum pose duration, thereby providing more focused view on larger time frames. In this way, poses based on short temporary spine movements can be omitted. On the other hand it is also possible to explore the movement on the chair, by exploring, e. g., a time frame from 0 to 10 seconds.

Another implemented filtering technique is the combination of timely-neighboring postures. Different bar charts of the same type might succeed each other. If the user wants to ignore the fact that these postures were interrupted by body movement (here: with each a duration of less than 10 seconds), it is possible to merge these neighboring postures.

**Fig 8:**
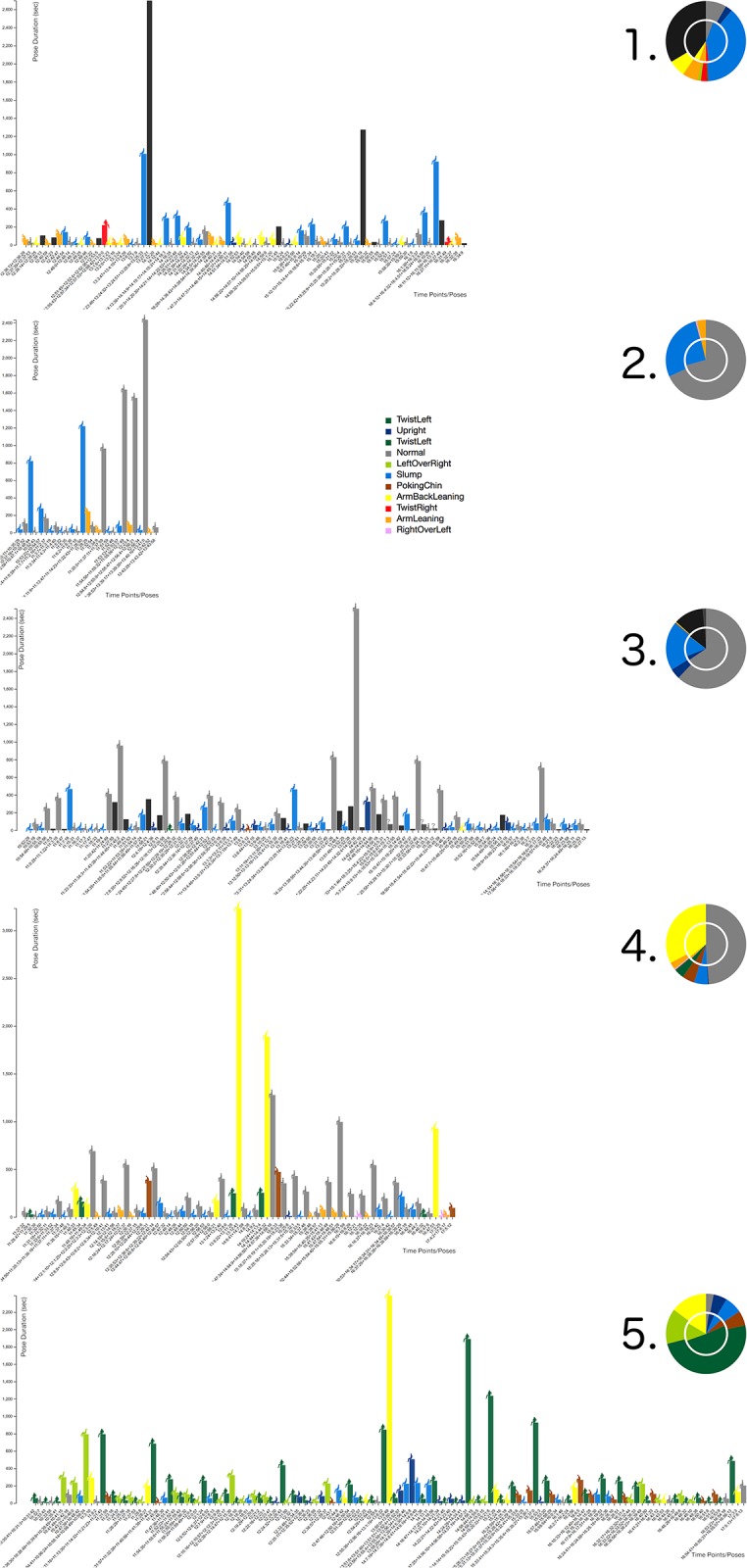
VSP-ADV sitting posture charts: Time lines. A high-resolution image of all postures with a duration *d* > 10 *sec*. ordered by starting time. Working day-specific sitting posture patterns can be evaluated. See Fig 10 for posture X labels.

Furthermore, two scale modes were implemented: a) scaling to the actual website (HD resolution), or b) scaling along the X and Y axis in a way maintaining the readability of the bar chart by using a predefined size for each bar. In this way it is possible to use the same scale in case different posture charts should be compared, such as in Fig 8.

**Fig 9:**
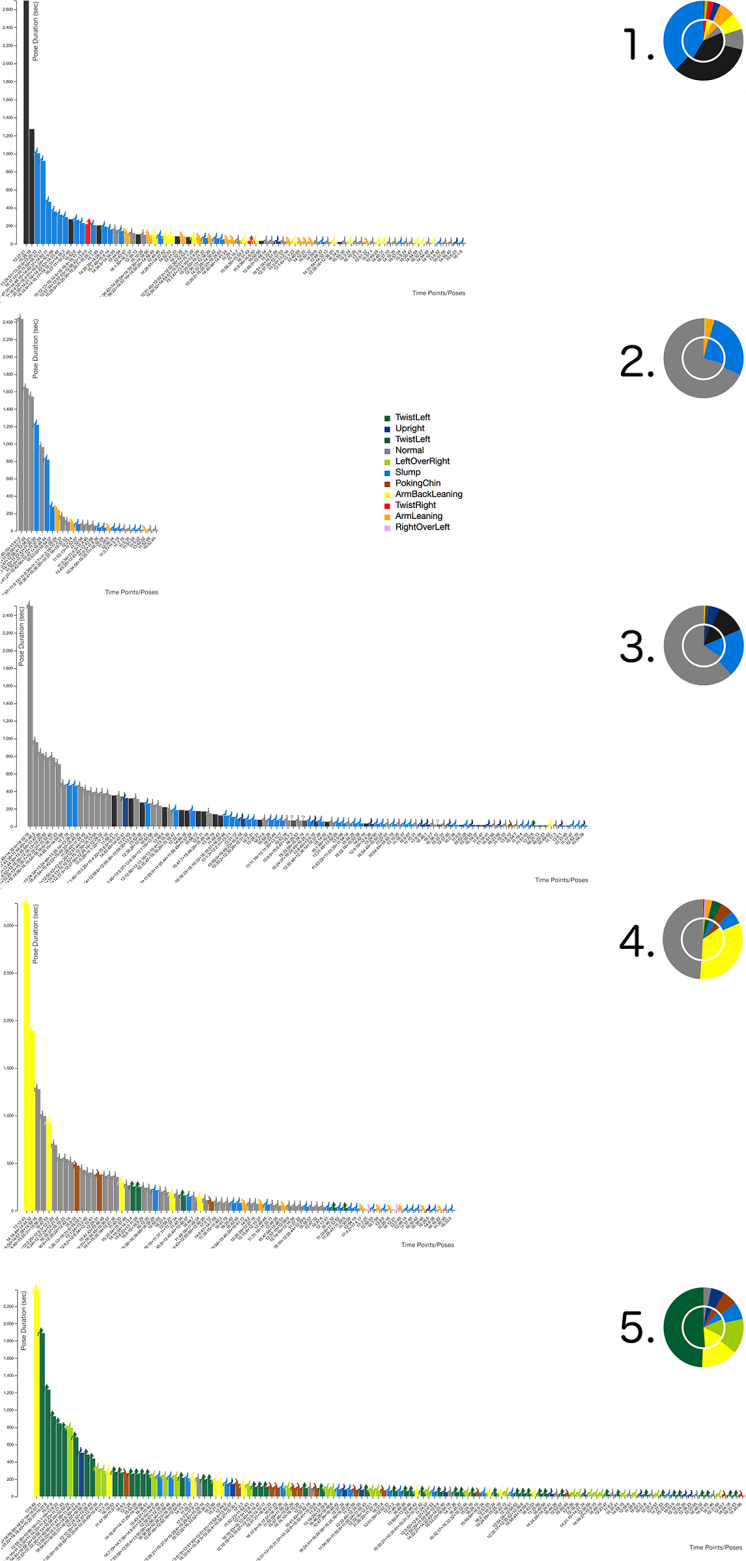
VSP-ADV sitting posture charts: Posture patterns. A high-resolution image of all postures with a duration *d* > 10 *sec*. ordered by pose duration. Subject-specific sitting posture patterns can be evaluated.

**Fig 10:**
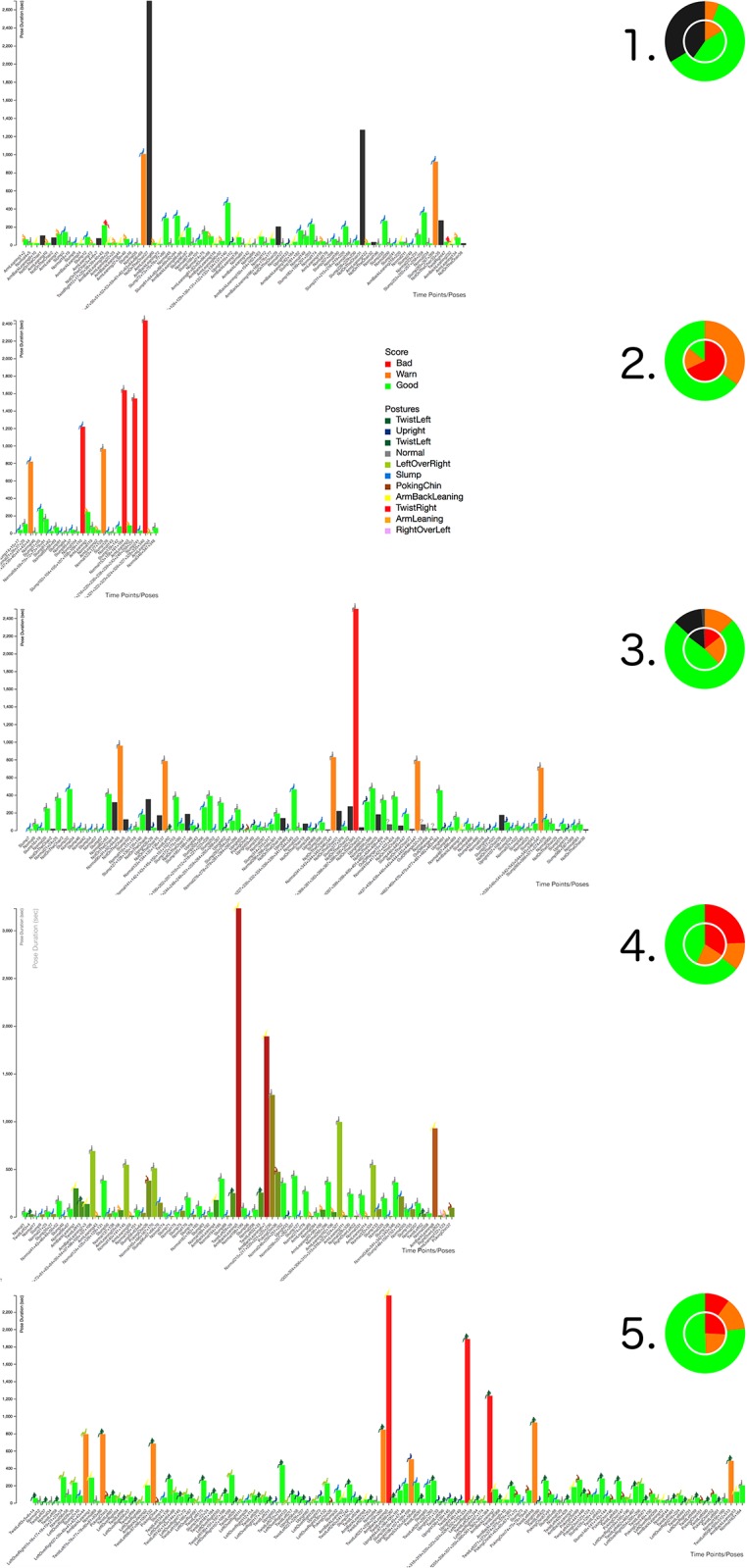
VSP-ADV sitting health posture charts: Time lines. A high-resolution image of all postures with a duration *d* > 10 *sec*. ordered by starting time. The duration-related rating is shown here. See Fig 8 for time X labels.

Another interaction technique is sorting: along the X-axis, the pose bars can be sorted a) according to the time sequence (Figs 8 and 10), or b) according to the pose duration (Fig 9). Switching between both states is possible in an animated fashion, enabling the user to follow the transition between both states.

*3. Details on Demand:* as the standard view does not show any numbers for the purpose of clarity, it is possible to mouse click (or using a smart phone: finger tap) at every bar chart or pie chart segment to see the concrete data: posture type, with its starting time and duration. In addition, the VSP-ADV system provides access to the complete data set. The selected data can be downloaded via the system and directly opened with, e. g., MS Excel. In addition, it is possible to show the posture names and IDs along the X axis (reflecting the timely sequence of the poses of the same type), or the corresponding time points.

To enable data analysis and online comparisons, the discussed system was implemented as a dynamic website based on HTML5, JavaScript, and D3 (Data-Driven Documents) [60].

Overall, the discussed VSP-ADV system uses five out of eight visual variables, as introduced by Ward et al. [61]: position, shape/mark, size, color (two color scales), and motion (sorting animation).

The user can select each of the five data sets which are discussed here. Fig 5 shows an example from the VSP-ADV online application: the visualized poses are filtered and show only those ones with a duration *d* of 60 *sec*. < *d* < 1,000,000 *sec*. Along the X-axis the poses are ordered based on the time sequence. The website as well as the data are available at: http://virtual-spine.immersive-analytics.org.

In addition, the Supporting Material S1 Websites provides the direct web links to the corresponding VSP-ADV visualizations discussed here.

### 2.6 Validation experiments

To validate the recognition of different sitting postures, an experiment with five participants was conducted. The goal was to better understand usage patterns and reactions to the application, which would indicate that VSP might be effective in the future in analyzing and changing sitting behaviors.

The collected data was anonymized. The voluntary participation in the study includes five male and female young-middle age adults (20-50 years), and a withdrawal was possible during the whole experiment. However, none of the subjects discussed in this study withdraw. Before the study started, a researcher contacted the potential participants to determine whether they are eligible to be included in the study. In this way it was avoided that, e. g., people with a history of complicated spinal pain or pregnant persons were involved in the study.

All participants provided written consent for reusing the data in future publications. The supplementary video does not show the actual experiment used to collect data for this work—it is showing the test period of the underlying methods. No personal data like age, sex, etc. was used in the context of this work, therefore the direct linkage to persons is not possible. However, all subjects shown on the video material were asked for their consent prior recording. The human ethics approval for the discussed experiment was achieved from the Monash University Human Research Ethics Committee (MUHREC) with the project number CF14/1137-2014000496.

#### Test period

At the beginning of the experiment the mat with the sensors was placed on a conventional office chair, and the subject was asked to complete a series of sitting positions on the chair during a period around 20 minutes (the time is not critical in this phase).

#### Initial recording

After this testing period, subjects returned to their regular work. The *Smart Chair* device—a conventional office chair equipped with the VSP-RTM sensors—was placed at the subject’s working place.

It is worth noting that the VSP-RTM module did not restrict the subject’s normal activities at the working place. During the initial recording, the subject was observed by our researchers to be able to define the different sitting postures in the following step. The Supporting Material provides the original data analyzed in the following sections, see Supporting Material S1 Data of Validation Experiments.

#### Calibration

Based on the data collected during the initial recording, VSP was configured so that it was able to recognize subject’s postures from the pressures applied during sitting. For this purpose, the previously discussed VSP-RTV method was used (2.4 Real-time Data Visualization). Fig 4, for example, shows a pressure pattern of a specific subject which is applied to the *Normal* pose.

#### Recording with visual feedback

Finally, a second part of the study was arranged with the subject using the Smart Chair with the regular working environmental setting. This time, visual feedback was provided to the user by the VSP-RTV method. The purpose was to encourage the subjects to have a balanced posture and to intermittently get up from sitting. This time the Smart Chair was used over the whole working day.

## 3. Results

To analyze different sitting postures, an initial experiment was conducted involving five subjects. The goal was to better understand usage patterns and reactions to the application which would indicate that VSP might be effective in the future in analyzing and changing sitting behaviors. As previously discussed, only postures with a duration of *d* > 10 *sec*. were taken into account.

The analysis of the data shows that partly extreme differences between the five subjects are visible. This brings us back to the initial statement that chronological sequences of individual sitting postures during the day are as unique as fingerprints. Examining each posture separately, it is possible to get a good overview of the movement during the course of the day. Additionally, these sitting posture charts can be used to compare and analyze the posture patterns of different subjects: Figs 8 and 9 use the posture type-encoding color scale, and Fig 10 the posture health rating one. Each representation provides a different analytical view at the posture patterns over the working day: The posture bar charts in Fig 8 provide the best overview which postures were used and at which times the user made a break during the working day. Fig 9 enables a fast glance at the priorities of the different postures over the day, ordered by their posture duration time. Fig 10 follows the same approach by using the health score posture color codes. This approach provides significant support to determine whether the posture durations were problematic or healthy.

The pie chart has been considered as the the best method for the global comparison and judgment of the five sitting postures. Starting from the pie charts in Fig 10, the users’ sitting behaviors can be quickly classified: subject 1 shows the best sitting behavior with no bad rating at all and the lowest warning rating.

Additionally, subject 1 had left the chair from time to time, as suggested by Ryan et al. [58]; here encoded with black color. Subject 3 comes next, due to relatively low warning and bad scores, additionally the subject had also occasionally left the chair, whereas all remaining subjects had remained in the chair over the complete measurement. Subject 5 comes next, followed by 4, both presenting a quite similar rating result (see pie chart). The worst behavior was shown by subject 2. Interestingly, the inner pie chart—which represents the filtered data with a minimal duration of 10 seconds—differs drastically from the summary of the complete data shown by the outer circle. This means that the subject was maintaining postures over a relatively long time even if every short posture change is taken into account, which represents a comparatively inflexible sitting behavior.

Whereas the health rating color codes are the first choice for the global comparison of the different subject’s sitting behavior, the posture-based color coding enables their in-detail analysis. The best sitting behavior patterns within these five subjects was presented by subject 1 with a regularly-changed sitting pattern. The postures *Normal* and *Upright* are only maintained for a short period. It is worth noting that this subject had left the chair for two long periods (around 2 p.m. for approx. 40 min.), which helped to increase the overall rating as well. Different to subject 1, subject 2 was sitting in *Slump* pose most of the time. Although subject 2 was shifting between different poses at the beginning, there was a long period when the subject was only in the *ArmBackLeaning* pose. After a break around 4 p.m., again the slump pose dominated the subject’s sitting pattern.

While the health-related sitting behavior ratings for subjects 2 and 3 were quite distinct to each other, it can be seen that the postures they used are quite similar. Both frequently use the *Normal* posture and from time to time the *Slump* posture. However, the rating difference comes from the extended posture duration of subject 2 and the fact that it remains on the chair. Certainly, it is also important to note that subject 2’s working period was nearly twice to subject 3. It is interesting to notice the change in subject 2’s behavior. He/she primarily used the *Slump* posture, then it changed to the *Normal* posture. Like the previous subjects, Subject 4 used the *Normal* posture quite often, but the second most often used posture was *ArmBackLeaning* which was maintained for ca. 50 min after 1:00 p.m. The reason for this behavior pattern could be: the subject a) was relaxing for a long time on the chair after lunch, b) took a nap, or c) had a longer discussion with colleagues. The reader should also keep in mind that short posture changes of a duration less than 10 seconds were filtered out and only regarded as minor movements.

As subject 5 indicates in Fig 9 it is important to evaluate the sitting postures in detail. A number of problematic aspects are visible here: firstly, the subject has the longest working day and never leaves the chair, secondly, the posture *TwistLeft* was used for more than half of the working day (see pie chart Fig 9.5). Therefore, it can be expected that the configuration of the workspace required the subject to frequently turn towards another object or person, e. g., the subject was using two computers at the same time (like it is the case in Fig 1a). This setting should be changed as it is not healthy to use this certain posture for a long time.

For detailed information regarding the overall duration and the direct links to the sitting posture charts with the corresponding time filters applied, please refer to the Supplementary Section Supporting Information.

## 4. Discussion

Even though there are methods available to monitor spinal health-related body movements, however, they require attachment to the user’s body or are limited to lab environments. Furthermore, none of the existing approaches provide practical solutions for daily monitoring needs, due to reasons such as a) it is often time-consuming, b) it may not allow assessment of the subject in a normal environment setting, and c) they are often impractical as a solution for longer time periods or large groups of persons.

As an alternative solution intended to deal with the challenges mentioned above, we presented here the design and implementation of the Virtual-Spine Platform (VSP). For a first evaluation, an Accumulative Data Visualization method was developed, which provides a practical, interactive solution to analyze the spinal health related sitting behavior in everyday environments—such as the home or workplace—and to analyze and compare specific sitting posture patterns.

To evaluate our new methodology, an expert questionnaire was conducted. Four of the five original participants were available for this small study. We collected data regarding their sitting behavior: two participants were sitting between 3-6 hours, one for 6-9 hours, and one more than 9 hours a day. All participants were regularly using the Internet during their daily work. Asked for their sitting behavior, one was rating the behavior as “somewhat positively”, two as “neither nor”, and one as “negatively”. Asked for potential back pain, one answered “no”, one answered “weak” and two answered “somewhat strong”. Summing up, it can be stated that each of the participants are part of the target group of VSP, spending a large amount of time sitting in front of the computer, resulting partly in back pain.

**Fig 11:**
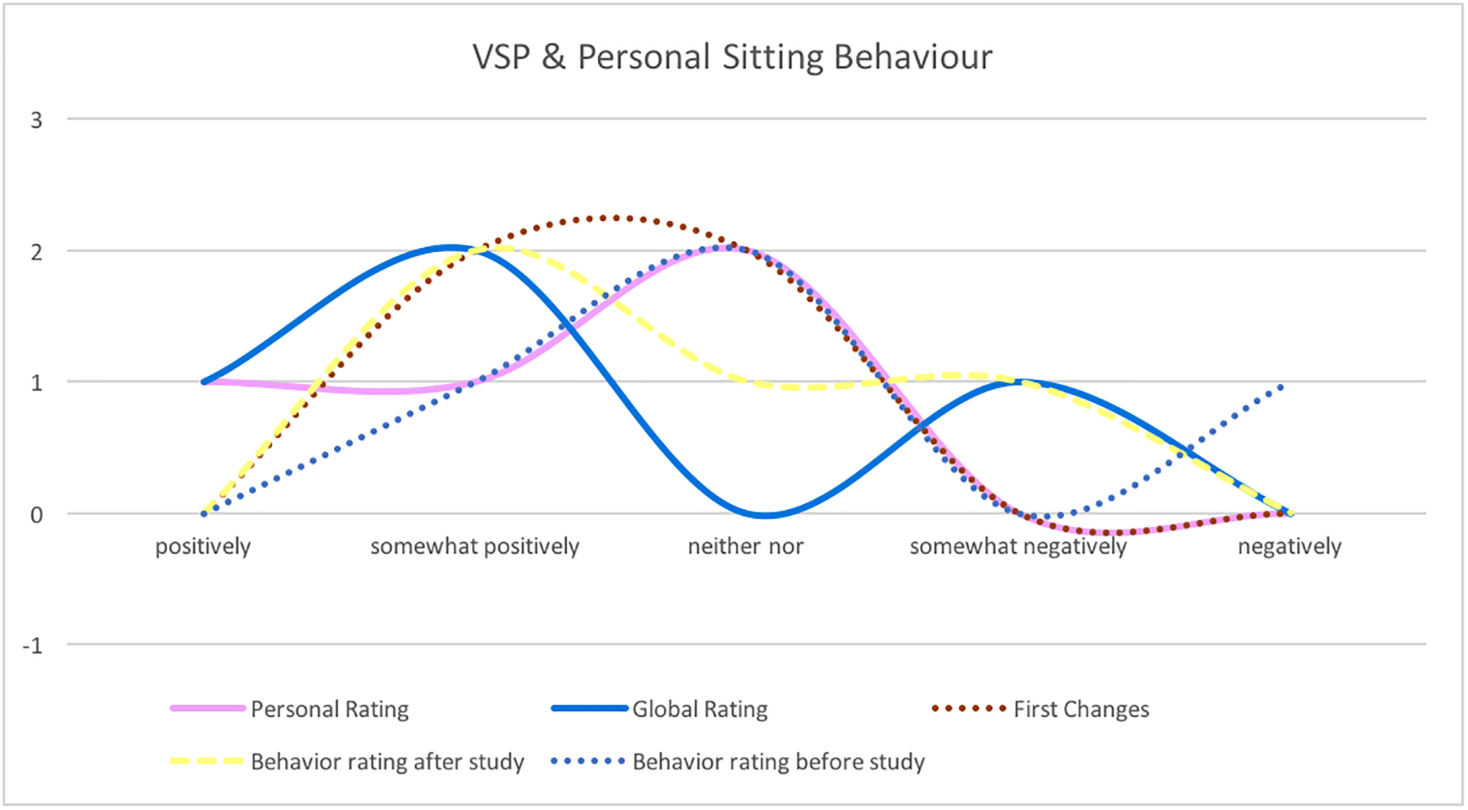
VSP personal sitting behavior. Personal Rating, Global Rating, First Changes, Sitting Behavior rating before and after the study.

Fig 11 shows the rating according the participant’s sitting behavior and the expected impact of VSP based on the following questions: *Personal Rating:* Do you think the Virtual Spine might have a positive impact on your personal life? *Global Rating:* Do you think the Virtual Spine might have a positive impact on the global community? *First Changes:* Did you already change your sitting behavior after using Virtual Spine during the initial experiment some time ago? *Behavior rating before study:* How do you rate your personal sitting behavior? *Behavior rating after study:* After using Virtual Spine ADV for the first time, how do you rate your personal sitting behavior? The ratings show that the participants expect a positive change in their sitting behavior by using VSP and that VSP was able to improve the consciousness regarding their sitting behavior. However, a single person was not convinced that VSP might have a positive impact on her/his sitting behavior.

**Fig 12:**
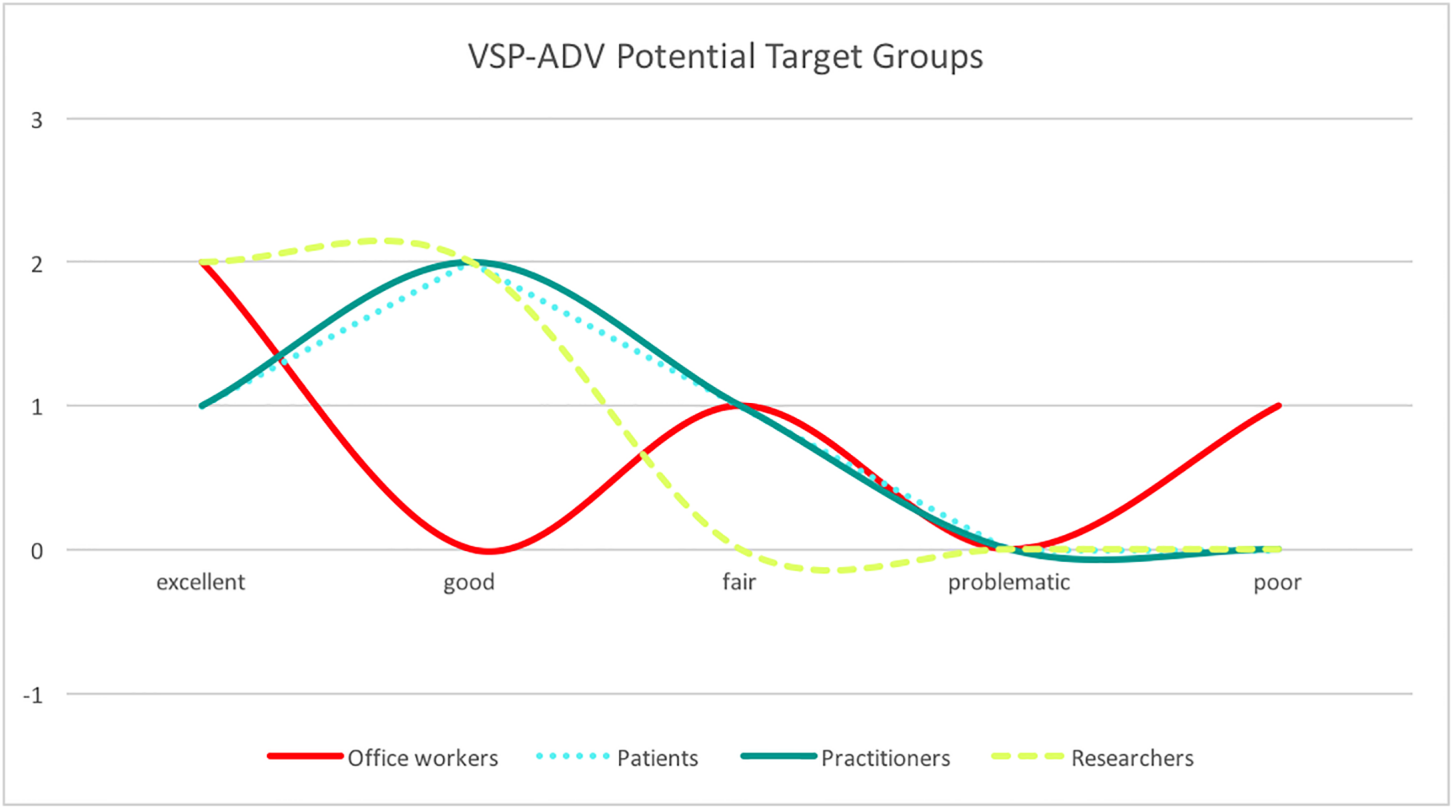
VSP-ADV potential target groups. Potential target groups.

The participants were also asked which potential target groups might be interested in using VSP and how useful VSP might be for those groups. Fig 12 shows the results: the participants expect VSP to be a great tool for research purposes, practitioners and patients, however, one person was not so convinced regarding the usefulness for office workers, maybe because of the prototype character of VSP.

**Fig 13:**
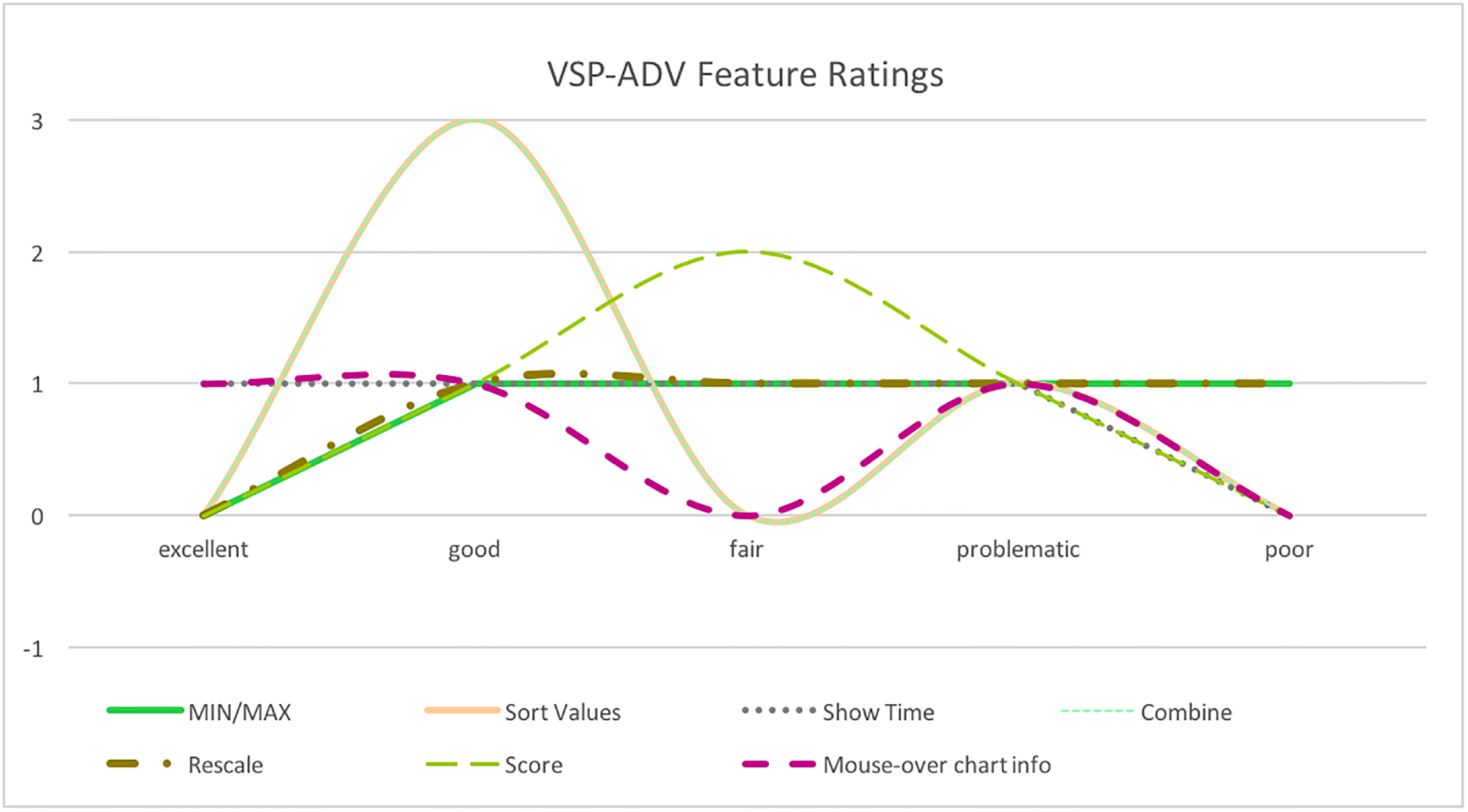
VSP-ADV feature ratings. Ratings of the different features part of VSP-ADV.

**Fig 14:**
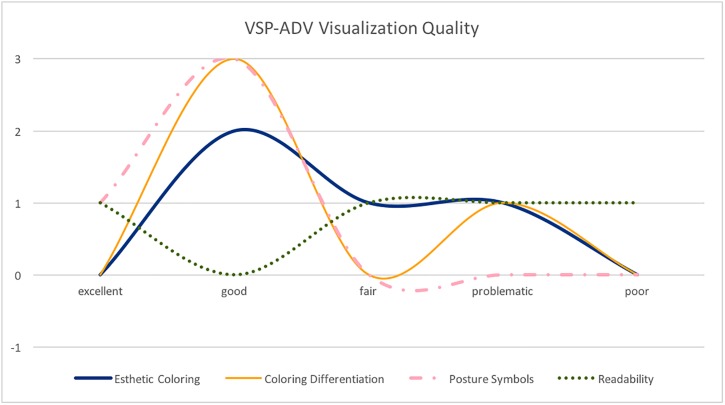
VSP-ADV visualization quality. Visual Quality of VSP-ADV: Esthetic Coloring, Coloring Differentiation, Posture Symbols, and Readability.

Next, the participants were asked to evaluate the VSP-ADV system. Fig 13 shows the result of the evaluation of the features of VSP-ADV, and Fig 14 elaborates the quality of the visualization. The following two features got the best ratings: *Sort values* and *Combine*. *Sort values:* in Fig 8 the sitting postures are unsorted, in Fig 9 they are sorted based on their duration. *Combine:* this feature combines neighboring postures of the same type. *Show Time:* Fig 8 shows the time as labels, and Fig 10 shows the posture names as labels—this feature received averagely good ratings. *MIN/MAX:* changes the minimum and maximum duration of the shown postures. In Fig 5 *MIN* is 60 sec. and *MAX* is set to an arbitrary large value. This feature got both positive as well as negative ratings. A potential reason might be that users expect here an automatized way to filter the values. *Rescale:* the user can change the width and height of the chart. This feature was only requested by two persons. *Score:* Fig 6 shows the scoring posture-dependent coloring, judging the length of the posture duration. The scoring feature got average ratings; the idea to rate the posture durations is only partly well-perceived. *Mouse-over chart info:* Fig 5 shows the mouse-over information listing details of the selected posture and received two positive ratings and one negative one (one participant did not provide an opinion).

Finally, the participants were asked to rate the visualization quality of VSP-ADV. Fig 14 shows that it got overall good ratings. Exclusively good ratings received the posture symbols. In addition, the color differentiation and aesthetic coloring were positively judged. Mixed ratings were obtained for the readability. Although there is a feature to manually rescale the size of the posture panels, the readability should be improved in the future.

Whereas three of four users were quite convinced by VSP as well as the VSP-ADV approach, a single user had a more negative opinion. Here, collected feedback is provided, which should be taken into account during the future development of VSP as well as related approaches:

1. The experiment did not fully translate to personal sitting habits. VSP should be tested on different types of chairs, as it can be expected that different persons respond with different postures when they are seated on different designs, reflecting personal habits or lifestyle.
2. VSP raised some awareness of the importance of ‘better’ postures but it does not offer corrective solutions for subjects wanting more beneficial feedback. The visualization should take different body parts into account to help subjects identifying problematic regions.
3. The posture rating based on the duration was not straight forward for all users. In the future, their might be more specific ratings, based on, e.g., posture-associated advices from practitioners.
4. On some computers, there were problems with the scaling of the VSP-ADV applications. Although there is a manual scaling feature implemented, users might prefer and optimized scaling functionality compatible across all platforms.
5. Moreover, in the future there should be different versions of the software, optimized to the needs of specific user groups and formats, attracting long term use and data collection.

## 5. Conclusions

VSP can be used to gain insights into the potential benefits of rehabilitations for people with current or past back pain, such as developing recommendations for type and timing of mild stretching activity prompts during a sedentary day. For this purpose, the sitting posture charts were developed, providing different ways to visualize the sitting behavior following Shneiderman’s visualization mantra: Overview first, Zoom and Filter, and Details on Demand [46]. Whereas the pie charts give a quick overview regarding the personal ranking of sitting postures, the bar chart can be used to examine the chronology of sitting postures. To enable a quick interpretation of the results, we combined simple 2D displays (charts) with an Iconic display (posture icons) [47].

The proposed system can be used to investigate

- how sitting influences spinal postures,
- how posture changes with durations of sitting and intermittent activity,
- how long subjects can/will remain in a certain posture,
- how often subjects change their sitting positions,
- how often repeated posture changing patterns occur, e. g. *a* to *b* to *a*, and
- the impact of the predefined position thresholds.

The VSP platform provides a strong potential to positively impact on daily sitting behaviors through direct and real-time as well as accumulated feedbacks while sitting on the chair.

This was shown by an initial expert study which evaluated the quality of the visualization as well as helped to estimate the future impact for changing the personal sitting behavior as well as to trigger global change in sitting behavior. Whereas three of four participants had a positive opinion towards VSP/VSP-ADV, a single person was more critical but provided valuable feedback for future developments with a specific focus on personalized corrective solutions.

In this study, due to the lack of a unified understanding of ‘good’ or ‘bad’ sitting postures in current clinical beliefs, the experiment mainly focused on two elements: ‘time’ and ‘repeated position patterns’. In the next stage, VSP could be designed to provide immediate warnings in case the duration of a pose is too long, changing body positions with too little movements, or changing with repeated patterns. Various warning types and different medias for communicating those warnings could be examined. In the future, based on a better definition of the ‘ideal’ sitting postures, the VSP “Snap Shot” positions could also be defined based on the different healthy levels (e. g. *Sitting Straight Up* >*Slump*). Also, the appropriate range of sitting time for various positions could provide the user with more accurate immediate sitting behavior advice. Furthermore, another extension would be more immersive visualization approaches combined with interactive analytics [62] to allow users, (distant) doctors and other health-related experts to better explore data regarding user’s sitting behavior, to identify sitting behavior dangerous for the back. While the improvement of sitting behavior has obvious advantages for the user or patient, it might also have benefits for health insurances which can acknowledge good sitting behavior and decrease their treatment costs for spine-related diseases.

The Virtual-Spine project website with additional information is located at http://www.virtual-spine.org.

## Supporting information

S1 Video VSP-RTV pose recording. The video shows the use of the VSP-RTV. Pose patterns are recorded over time with different subjects.
(MP4)

S1 Websites VSP website and VSP-ADV website with sitting posture charts weblinks. Virtual Spine Website with additional information for the discussed VSP project.Sitting Posture Weblinks based on the link to the web application discussed in 2.5 Accumulated Data Visualization for the charts shown on Figs 5 and 6 with a duration of > 60 seconds, and Figs 8–10 with a duration of > 10 seconds.
(PDF)

S1 Data of Validation Experiments CSV data of 5 subjects. The data collected during the validation experiments of five subjects in CSV format discussed in 2.6 Validation Experiments.
(ZIP)

## References

[1] Vos T, Flaxman AD, Naghavi M, Lozano R, Michaud C, Ezzati M, et al Years lived with disability (YLDs) for 1160 sequelae of 289 diseases and injuries 1990–2010: a systematic analysis for the Global Burden of Disease Study 2010. The Lancet. 2013;380(9859):2163–2196. doi: 10.1016/S0140-6736(12)61729-2 10.1016/S0140-6736(12)61729-2 PMC6350784 23245607

[2] Bauman A, Ainsworth BE, Sallis JF, Hagströmer M, Craig CL, Bull FC, et al The descriptive epidemiology of sitting: a 20-country comparison using the International Physical Activity Questionnaire (IPAQ). American Journal of Preventive Medicine. 2011;41(2):228–235. doi: 10.1016/j.amepre.2011.05.003 21767731 10.1016/j.amepre.2011.05.003

[3] Roffey DM, Wai EK, Bishop P, Kwon BK, Dagenais S. Causal assessment of occupational sitting and low back pain: results of a systematic review. The Spine Journal. 2010;10(3):252–261. doi: 10.1016/j.spinee.2009.12.005 20097618 10.1016/j.spinee.2009.12.005

[4] Brink Y, Louw QA. A systematic review of the relationship between sitting and upper quadrant musculoskeletal pain in children and adolescents. Manual therapy. 2013;18(4):281–288. doi: 10.1016/j.math.2012.11.003 23298827 10.1016/j.math.2012.11.003

[5] Cohen D. An objective measure of seat comfort. Aviation, Space, and Environmental Medicine. 1998;69(4):410–414. 9561290

[6] Helander MG, Zhang L. Field studies of comfort and discomfort in sitting. Ergonomics. 1997;40(9):895–915. doi: 10.1080/001401397187739 9306741 10.1080/001401397187739

[7] Monette M, Weiss-Lambrou R, Dansereau J. In search of a better understanding of wheelchair sitting comfort and discomfort. In: Proc. of the RESNA Conf.; 1999. p. 218–220.

[8] Shaw G. Wheelchair seat comfort for the institutionalized elderly. Assistive Technology. 1991;3(1):11–23. doi: 10.1080/10400435.1991.10132176 10149067 10.1080/10400435.1991.10132176

[9] Bergstrom N, US Dept of Health and Human Services. Pressure ulcers in adults: prediction and prevention. Rockville, Maryland, USA: Agency for Health Care Policy and Research, Public Health Service, US Department of Health and Human Services; 1992.

[10] Brandeis GH, Ooi WL, Hossain M, Morris JN, Lipsitz LA. A longitudinal study of risk factors associated with the formation of pressure ulcers in nursing homes. Journal of the American Geriatrics Society. 1994;42(4):388–393. doi: 10.1111/j.1532-5415.1994.tb07486.x 8144823 10.1111/j.1532-5415.1994.tb07486.x

[11] Berlowitz D, Wilking S. Pressure ulcers in the nursing home. Improving care in the nursing home: comprehensive reviews of clinical research. 1993;4:102–30. doi: 10.4135/9781483325903.n4

[12] Burnett AF, Cornelius MW, Dankaerts W, O’Sullivan PB. Spinal kinematics and trunk muscle activity in cyclists: a comparison between healthy controls and non-specific chronic low back pain subjects—a pilot investigation. Manual Therapy. 2004;9(4):211–219. doi: 10.1016/j.math.2004.06.002 15522646 10.1016/j.math.2004.06.002

[13] Gleckler AD, Ng VV, Whitworth D, Cotner K, Hall TR. Providing information related to the posture mode of a user applying pressure to a seat component; 2013.

[14] Koskelo R, Vuorikari K, Hänninen O. Sitting and standing postures are corrected by adjustable furniture with lowered muscle tension in high-school students. Ergonomics. 2007;50(10):1643–1656. doi: 10.1080/00140130701587236 17917904 10.1080/00140130701587236

[15] Corlett E. Background to sitting at work: research-based requirements for the design of work seats. Ergonomics. 2006;49(14):1538–1546. doi: 10.1080/00140130600766261 17050393 10.1080/00140130600766261

[16] Chavalitsakulchai P, Shahnavaz H. Ergonomics method for prevention of the musculoskeletal discomforts among female industrial workers: physical characteristics and work factors. Journal of Human Ergology. 1993;22(2):95–113. 7963485

[17] Faust E, Pfahler K, Schmidt H. Method for quantitative determination of the pressure comfort of a seat cushion; 1999.

[18] Podoloff RM. Automotive seating analysis using thin, flexible tactile sensor arrays. SAE Technical Paper; 1993.

[19] Gleckler AD, Ng VV, Whitworth D, Cotner K, Hall TR. Providing information related to the posture mode of a user applying pressure to a seat component; 2013.

[20] Breed DS, Johnson WC, DuVall WE. Arrangement for sensing weight of an occupying item in vehicular seat; 2014.

[21] Vos-Draper TL, Rindflesch A, Morrow MM. Wireless, Real-Time Seat Interface Pressure Mapping With a Smartphone as Biofeedback For Positioning and Pressure Relief. In: Proc. of the RESNA Conf.; 2013.

[22] Gadotti I, Magee D. Validity of surface markers placement on the cervical spine for craniocervical posture assessment. Manual Therapy. 2013;18(3):243–247. doi: 10.1016/j.math.2012.10.012 23158022 10.1016/j.math.2012.10.012

[23] Straker LM, O’Sullivan PB, Smith AJ, Perry MC. Relationships between prolonged neck/shoulder pain and sitting spinal posture in male and female adolescents. Manual Therapy. 2009;14(3):321–329. doi: 10.1016/j.math.2008.04.004 18555730 10.1016/j.math.2008.04.004

[24] Spielholz P, Silverstein B, Morgan M, Checkoway H, Kaufman J. Comparison of self-report, video observation and direct measurement methods for upper extremity musculoskeletal disorder physical risk factors. Ergonomics. 2001;44(6):588–613. doi: 10.1080/00140130118050 11373023 10.1080/00140130118050

[25] O’Sullivan K, Clifford A, Hughes L. The reliability of the CODA motion analysis system for lumbar spine analysis: a pilot study. Physiotherapy Practice and Research. 2010;31(1):16–22.

[26] O’Sullivan K, Galeotti L, Dankaerts W, O’Sullivan L, O’Sullivan P. The between-day and inter-rater reliability of a novel wireless system to analyse lumbar spine posture. Ergonomics. 2011;54(1):82–90. doi: 10.1080/00140139.2010.535020 21181591 10.1080/00140139.2010.535020

[27] Nuwer MR, Dawson EG, Carlson LG, Kanim LE, Sherman JE. Somatosensory evoked potential spinal cord monitoring reduces neurologic deficits after scoliosis surgery: results of a large multicenter survey. Electroencephalography and Clinical Neurophysiology/Evoked Potentials Section. 1995;96(1):6–11. doi: 10.1016/0013-4694(94)00235-D 10.1016/0013-4694(94)00235-d 7530190

[28] Nash J, Clyde L, Loring R, Schatzinger L, Brown R. Spinal cord monitoring during operative treatment of the spine. Clinical Orthopaedics and Related Research. 1977;126:100–105. 598095

[29] Nuwer MR. Spinal cord monitoring. Muscle & Nerve. 1999;22(12):1620–1630. doi: 10.1002/(SICI)1097-4598(199912)22:12%3C1620::AID-MUS2%3E3.0.CO;2-1 10567073 10.1002/(sici)1097-4598(199912)22:12<1620::aid-mus2>3.0.co;2-1

[30] Caihua W. Vertebra center detection apparatus using spinal-cord region detection, method and recording medium storing a program; 2012.

[31] Deletis V, Sala F. Intraoperative neurophysiological monitoring during spine surgery: an update. Current Opinion in Orthopaedics. 2004;15(3):154–158.

[32] Grundy BL. Intraoperative Monitoring of Sensory Evoked Potentials In: Anesthesiology 1986 Springer; 1986. p. 211–230. 10.1097/00000542-198301000-00011 6401201

[33] Calancie B, Harris W, Broton JG, Alexeeva N, Green BA. “Threshold-level” multipulse transcranial electrical stimulation of motor cortex for intraoperative monitoring of spinal motor tracts: description of method and comparison to somatosensory evoked potential monitoring. Journal of Neurosurgery. 1998;88(3):457–470. doi: 10.3171/jns.1998.88.3.0457 9488299 10.3171/jns.1998.88.3.0457

[34] O’Sullivan PB, Burnett A, Floyd AN, Gadsdon K, Logiudice J, Miller D, et al Lumbar repositioning deficit in a specific low back pain population. Spine. 2003;28(10):1074–1079. doi: 10.1097/01.BRS.0000061990.56113.6F 12768152 10.1097/01.BRS.0000061990.56113.6F

[35] Donatell GJ, Meister DW, O’Brien JR, Thurlow JS, Webster JG, Salvi FJ. A simple device to monitor flexion and lateral bending of the lumbar spine. Neural Systems and Rehabilitation Engineering, IEEE Transactions on. 2005;13(1):18–23. doi: 10.1109/TNSRE.2005.843446 10.1109/TNSRE.2005.843446 15813402

[36] Magnusson ML, Chow DH, Diamandopoulos Z, Pope MH. Motor control learning in chronic low back pain. Spine. 2008;33(16):E532–E538. doi: 10.1097/BRS.0b013e31817dfd9a 18628693 10.1097/BRS.0b013e31817dfd9a

[37] Sheeran L, Sparkes V, Busse M, van Deursen R. Preliminary study: reliability of the spinal wheel. A novel device to measure spinal postures applied to sitting and standing. European Spine Journal. 2010;19(6):995–1003. doi: 10.1007/s00586-009-1241-0 20013001 10.1007/s00586-009-1241-0 PMC2899977

[38] Mannion AF, Knecht K, Balaban G, Dvorak J, Grob D. A new skin-surface device for measuring the curvature and global and segmental ranges of motion of the spine: reliability of measurements and comparison with data reviewed from the literature. European Spine Journal. 2004;13(2):122–136. doi: 10.1007/s00586-003-0618-8 14661104 10.1007/s00586-003-0618-8 PMC3476568

[39] Spielman SB. Apparatus for monitoring spinal motion; 1995.

[40] O’Sullivan K, O’Sullivan L, Campbell A, O’Sullivan P, Dankaerts W. Towards monitoring lumbo-pelvic posture in real-life situations: Concurrent validity of a novel posture monitor and a traditional laboratory-based motion analysis system. Manual Therapy. 2012;17(1):77–83. doi: 10.1016/j.math.2011.09.006 22015373 10.1016/j.math.2011.09.006

[41] Wang SJ, Yu D. Virtual-spine: the collaboration between pervasive environment based simulator, game engine (mixed-reality) and pervasive messaging. In: Pervasive Computing Technologies for Healthcare (PervasiveHealth), 2013 7th Intl. Conf. IEEE; 2013. p. 45–48.

[42] Callaghan V, Clark G, Colley M, Hagras H, Chin J, Doctor F. Intelligent inhabited environments. BT Technology Journal. 2004;22(3):233–247. doi: 10.1023/B:BTTJ.0000047137.42670.4d

[43] Billinghurst M, Kato H. Collaborative mixed reality. In: Proc. Intl. Symp. Mixed Reality; 1999. p. 261–284.

[44] Wang SJ. Fields Interaction Design (FID): The answer to ubiquitous computing supported environments in the post-information age. Homa & Sekey Books; 2013.

[45] Claus AP, Hides JA, Moseley GL, Hodges PW. Is ‘ideal’sitting posture real?: Measurement of spinal curves in four sitting postures. Manual Therapy. 2009;14(4):404–408. doi: 10.1016/j.math.2008.06.001 18793867 10.1016/j.math.2008.06.001

[46] Shneiderman B. The eyes have it: A task by data type taxonomy for information visualizations. In: Proc. IEEE Symp. Visual Languages. IEEE; 2003. p. 336–343.

[47] Keim DA. Information visualization and visual data mining. IEEE Transactions on Visualization and Computer Graphics. 2002;8(1):1–8. doi: 10.1109/2945.981847

[48] Munzner T. Visualization analysis and design. CRC Press; 2014.

[49] Green-Armytage P. A colour alphabet and the limits of colour coding. JAIC—Journal of the International Colour Association. 2010;5:1–12.

[50] Sommer B, Kormeier B, Demenkov PS, Arrigo P, Hippe K, Ates Ö, et al Subcellular localization charts: a new visual methodology for the semi-automatic localization of protein-related data sets. Journal of Bioinformatics and Computational Biology. 2013;11(01):1340005 doi: 10.1142/S0219720013400052 23427987 10.1142/S0219720013400052

[51] Kovanci G, Ghaffar M, Sommer B. Web-based hybrid-dimensional Visualization and Exploration of Cytological Localization Scenarios. Journal of Integrative Bioinformatics. 2016;13(4):47–58. doi: 10.1515/jib-2016-298 28187414 10.2390/biecoll-jib-2016-298

[52] Nachemson A, Elfstrom G. Intravital dynamic pressure measurements in lumbar discs. Scand Journal Rehabilative Medicine. 1970;2(suppl 1):1–40. 4257209

[53] Nachemson AL. The Lumbar Spine An Orthopaedic Challenge. spine. 1976;1(1):59–71. doi: 10.1097/00007632-197603000-00009

[54] Wilke HJ, Neef P, Caimi M, Hoogland T, Claes LE. New in vivo measurements of pressures in the intervertebral disc in daily life. Spine. 1999;24(8):755–762. doi: 10.1097/00007632-199904150-00005 10222525 10.1097/00007632-199904150-00005

[55] Dublin P. Sitting Causes Back Pain—Physio Dublin—Laurel Lodge Physiotherapy, Dublin 15—01 8249585; 2015. Available from: http://www.physiodublin.ie/2015/11/17/sitting-causes-back-pain/.

[56] University G. Safe Lifting Techniques—Gonzaga University; 2017. Available from: http://www.gonzaga.edu/Campus-Resources/Offices-and-Services-A-Z/Human-Resources/Environmental-Health-And-Safety/Ergonomics/Safe-Lifting-Techniques.asp.

[57] Reenalda J, Van Geffen P, Nederhand M, Jannink M, IJzerman M, Rietman H. Analysis of healthy sitting behavior: Interface pressure distribution and subcutaneous tissue oxygenation. Journal of Rehabilitation Research and Development. 2009;46(5):577–586. doi: 10.1682/JRRD.2008.12.0164 19882492 10.1682/jrrd.2008.12.0164

[58] Ryan CG, Dall PM, Granat MH, Grant PM. Sitting patterns at work: objective measurement of adherence to current recommendations. Ergonomics. 2011;54(6):531–538. doi: 10.1080/00140139.2011.570458

[59] of Physiotherapy CS. FIT to WORK exercises; 2005. Available from: http://www.webcitation.org/6tKjfBwb9.

[60] Bostock M, Ogievetsky V, Heer J. D^3^ data-driven documents. IEEE Transactions on Visualization and Computer Graphics. 2011;17(12):2301–2309. doi: 10.1109/TVCG.2011.185 22034350 10.1109/TVCG.2011.185

[61] Ward MO, Grinstein G, Keim D. Interactive data visualization: foundations, techniques, and applications. CRC Press; 2010.

[62] Chandler T, Cordeil M, Czauderna T, Dwyer T, Glowacki J, Goncu C, et al. Immersive Analytics. In: IEEE Big Data Visual Analytics (BDVA) 2015. IEEE eXpress Conference Publishing; 2015. p. 73–80.

