# Supplementary material for: The Virtual-Spine Platform—Acquiring, visualizing, and analyzing individual sitting behavior
Source: PLoS One. 2018 Jun 13;13(6):e0195670. doi: 10.1371/journal.pone.0195670 (PMC5999082; doi:10.1371/journal.pone.0195670)
Supplement: S1 Websites — Virtual Spine Website with additional information for the discussed VSP project.Sitting Posture Weblinks based on the link to the web application discussed in 2.5 Accumulated Data Visualization for the charts shown on Figs 5 and 6 with a duration of > 60 seconds, and Figs 8–10 with a duration of > 10 seconds. (PDF) [file pone.0195670.s002.pdf]

Supporting Information for The *Virtual-Spine* Platform - Acquiring, Visualizing, and Analyzing Individual Sitting Behavior

Stephen Jia Wang[1,2*], Björn Sommer[3,4¤], Wenlong Cheng[5], Falk Schreiber[3,4]

1 Department of Innovation Design Engineering, School of Design, Royal College of Art, London, UK
2 International Tangible Interaction Design Lab, Monash University, Melbourne, Victoria, Australia
3 Department of Computer and Information Science, University of Konstanz, Konstanz, Baden-Württemberg, Germany
4 Faculty of Information Technology, Monash University, Melbourne, Victoria, Australia
5 Faculty of Engineering, Monash University, Melbourne, Victoria, Australia

* Corresponding Author for Virtual Spine: stephen.wang@rca.ac.uk
¤ Corresponding Author for VSP-ADV/Web Visualization: bjoern@CELLmicrocosmos.org

Virtual Spine Websites

Virtual-Spine Website. This is the link to the official Virtual-Spine project website:
`http://www.virtual-spine.org`

VSP-ADV Website. This is the link to the web application discussed in Section 2.5 Accumulated Data Visualization:
`http://virtual-spine.immersive-analytics.org`

Sitting Posture Charts Weblinks/VSP-ADV Website.
Figs 5 and 6 with a duration of > 60 seconds and combined charts:

Subject 1:
`http://virtual-spine.immersive-analytics.org/index.html?file=0&combine=true&min=60&max=1000000&rescale=false&xscale=60&yscale=0.3`
Overall Duration: 238.30 min (Filtered: 143.78)

More on the next page ...

Figs 8-10 with a duration of > 10 seconds and combined charts:

Subject 1:
http://virtual-spine.immersive-analytics.org/index.html?file=0&combine=true&min=10&max=1000000&rescale=false&xscale=15&yscale=0.3
Overall Duration: 238.30 min (Filtered: 197.52)

Subject 2:
http://virtual-spine.immersive-analytics.org/index.html?file=1&combine=true&min=10&max=1000000&rescale=false&xscale=15&yscale=0.3
Overall Duration: 191.23 min (Filtered: 167.53)

Subject 3:
http://virtual-spine.immersive-analytics.org/index.html?file=2&combine=true&min=10&max=1000000&rescale=false&xscale=15&yscale=0.3
Overall Duration: 336.63 min (Filtered: 289.90)

Subject 4:
http://virtual-spine.immersive-analytics.org/index.html?file=3&combine=true&min=10&max=1000000&rescale=false&xscale=15&yscale=0.3
Overall Duration: 339.66 min (Filtered: 312.42)

Subject 5:
http://virtual-spine.immersive-analytics.org/index.html?file=4&combine=true&min=10&max=1000000&rescale=false&xscale=15&yscale=0.3
Overall Duration: 398.50 min (Filtered: 355.90)
